# P-585. Improvement in Time to Viral Suppression Pre/Post an Anti-Retroviral Treatment and Access to Services Intervention (ARTAS) for Emergency Department Patients

**DOI:** 10.1093/ofid/ofae631.783

**Published:** 2025-01-29

**Authors:** Thomas Ludden, Sam Parker, Michael Leonard, Jeremy Thomas, Dasia Jefferson, Jaylyn McLean

**Affiliations:** Atrium Health, Charlotte, North Carolina; Atrium Health, Charlotte, North Carolina; Carolinas Medical Center Atrium Health, Charlotte, North Carolina; Atrium Health, Charlotte, North Carolina; Atrium Health, Charlotte, North Carolina; Atrium Health, Charlotte, North Carolina

## Abstract

**Background:**

While the benefits of early entry into medical care are well-documented, more than one-third of people living with HIV and AIDS (PLWHA) who know their serostatus are not linked to medical care. Nearly 39 percent delay entry into care by 12 months, and 32 percent delay entry for more than 2 years. People do not link to medical care following an HIV-positive diagnosis for a variety of reasons. These may include but are not limited to personal barriers such as unemployment, lack of health insurance, fear, stigma, substance use, mental health issues, lack of transportation, homelessness, and/or not having the required forms of identification to receive services. Other system-level barriers include wait lists for services and/or medications and complex, confusing administrative processes. Rarely do people face only one barrier to accessing medical care. Anti-retroviral treatment and Access to Services Intervention (ARTAS) is an individual-level, multi-session, time-limited intervention designed to link individuals who have been recently diagnosed with HIV in the Emergency Department (ED) to medical care.

**Methods:**

African American or Hispanic/Latino male patients who tested positive for HIV for the first time in the emergency department in an urban Southeastern location were included in the analysis. The number of days to viral suppression was calculated from the date of diagnosis to the undetectable viral load test. The average number of days was summarized and compared using the difference in means one year pre/post the implementation of ARTAS in the emergency department.

**Results:**

ARTAS was implemented on March 1, 2022. Eight patients were diagnosed with HIV in the ED one year before this date, with an average of 264 days to viral suppression. 53 patients were diagnosed one year after that date, with the average number of days from diagnosis to viral suppression decreased to 113 with an average difference of 151 days ([95%] CI 54.6150 to 247.3850, P=0.03). From the time of the first visit with an HIV provider to viral suppression was 86 and 85 days, respectively.

**Conclusion:**

An ARTAS implementation in the ED reduced the time from diagnosis to viral suppression by 57%.

**Disclosures:**

**All Authors**: No reported disclosures

